# Evaluating anti-GPL-core IgA as a diagnostic tool for non-tuberculous mycobacterial infections in Thai patients with high antibody background

**DOI:** 10.1038/s41598-023-45893-8

**Published:** 2023-11-02

**Authors:** Varis Manbenmad, Apichart So-ngern, Ploenchan Chetchotisakd, Kiatichai Faksri, Manabu Ato, Arnone Nithichanon, Ganjana Lertmemongkolchai

**Affiliations:** 1 Research and Diagnostic Center for Emerging Infectious Diseases (RCEID), Department of Microbiology, Faculty of Medicine, Khon Kaen University, Khon Kaen, Thailand; 2 Department of Medicine, Faculty of Medicine, Khon Kaen University, Khon Kaen, Thailand; 3 Department of Mycobacteriology, National Institute of Infectious Diseases, Tokyo, Japan; 4 Department of Medical Technology, Faculty of Associated Medical Sciences, Chiang Mai University, Chiang Mai, Thailand

**Keywords:** Immunology, Microbiology, Health care, Medical research

## Abstract

Diagnosis of non-tuberculous mycobacterial (NTM) infection is difficult due to low sensitivity and time-consuming laboratory tests. Current serological assays fail in tropical countries due to high antibody background. This study aimed to investigate an appropriate method for detecting anti-glycopeptidolipid (GPL)-core antibodies to diagnose NTM infection in Thailand. Heparinized plasma samples were collected from 20 patients with NTM-pulmonary disease (NTM-PD) and 22 patients with disseminated NTM (dNTM) for antibody detection by ELISA. The results were compared with those from patients with tuberculosis, other bacterial pulmonary infections and healthy controls. Among the different antibody isotypes, anti-GPL-core IgA exhibited the highest suitability. Therefore, anti-GPL-core IgA and its subclass IgA2 were further investigated. A significant increase in antibody levels was observed during the active infection stage, whereas NTM-PD with culture conversion at the 6-month follow-up showed reduced IgA levels. The diagnostic cut-off for IgA and IgA2 was newly defined as 1.4 and 1.0 U/ml, respectively. Using our IgA cut-off, the sensitivity and specificity for diagnosing NTM-PD were 77.3% and 81.4%, respectively. The new IgA cut-off demonstrated significantly improved specificity compared to the manufacturer's cut-off. Thus, serological detection of anti-GPL-core IgA, with a cut-off of 1.4 U/ml, can be a valuable tool for supporting NTM diagnosis in Thailand.

## Introduction

Non-tuberculous mycobacteria (NTM) are commonly found in the environment, and their infections have become a global public health problem due to the increasing number of cases worldwide^1^. NTM infections can occur in both immunocompetent and immunocompromised patients^2,3^. The most common clinical manifestation is NTM pulmonary disease (NTM-PD), although disseminated NTM (dNTM) infections have also been reported^4^. Currently, the diagnosis of NTM-PD relies on chest radiography and positive NTM cultures from clinical specimens^5^. Bacterial culture is considered the gold standard for laboratory investigations; however, it has two major drawbacks: time consumption and lack of sensitivity^6^.

To improve the diagnostic efficiency of NTM infection, several molecular approaches have been introduced, such as polymerase chain reaction, multi-locus sequence typing, nucleic acid amplification tests, line probe assays, and next-generation sequencing. However, the overall sensitivity of these approaches is only 29–76%^7,8^. Moreover, serological diagnosis of NTM-PD, particularly infection with the Mycobacterium avium complex (MAC), has been introduced using the detection of IgA antibodies against a component on the surface of NTM called the glycopeptidolipid (GPL) core^9^. This assay is based on an ELISA and is simple with a high throughput. Several studies conducted in Japan, Taiwan, South Korea, and the United States have reported a sensitivity of approximately 60–90% and a specificity of 91–100%^9–13^. However, the distribution of NTM-infected cases in previous studies differs from that in Thailand. In Thailand, the distribution of NTM is non-MAC group-dominant, similar to that in China and other Southeast Asian countries^14^. A study on NTM infection in northeastern Thailand reported that the most common causative agent is *Mycobacterium abscessus*, a rapidly growing mycobacterium (RGM)^15^. Considering the differences in NTM distribution, the efficiency of the test kit should be evaluated in specific populations to substantiate its validity in Thailand and other countries with RGM dominance.

Another challenge when applying serological tests for diagnosing infectious diseases in tropical countries is the high background of antibodies. Therefore, this study aimed to (1) investigate an appropriate antibody isotype and the cut-off for Thai patients with NTM infection and (2) evaluate the diagnostic efficacy of plasma anti-GPL-core for Thai patients with NTM-PD and dNTM in comparison with *Mycobacterium tuberculosis* pulmonary disease (MTB-PD) and other bacterial pulmonary diseases. Our results highlight that anti-GPL-core IgA at a cut-off of 1.4 U/ml can be applied for the diagnosis and monitoring of Thai patients with NTM-PD or dNTM.

## Results

### Prevalence of *M. abscessus* in Thai patients with NTM pulmonary disease and disseminated NTM infection

Eighty-eight patients with bacterial infections and 25 healthy controls were enrolled. Bacterial culture-positive patients were classified into four groups: 20 with NTM pulmonary disease (NTM-PD), 22 with disseminated NTM infection (dNTM), 14 with *Mycobacterium tuberculosis* pulmonary disease (MTB-PD), and 32 with other bacterial pulmonary diseases (Oth-PD). The general demographic characteristics of the participants are presented in Table 1. The median age of the patients was 57 years, with an interquartile range of 16 years. Comparisons among the sample groups revealed a significant difference in age only between the MTB-PD and Oth-PD groups (P-value = 0.0097). The distribution of sex was not balanced, with varying proportions of males and females across the subgroups.

**Table 1:** General demographics of study participants.

	NTM-PD (n = 20)	dNTM (n = 22)	MTB-PD (n = 14)	Oth-PD (n = 32)	HC (n = 25)
Median of age in years (IQR)	60 (34–81)	54 (40–72)	47 (18–67)*	62 (38–86)*	53 (23–70)
Number of females (%)	14/20 (70.0%)	9/22 (40.1%)**	5/14 (35.7%)**	13/32 (40.6%)**	16/25 (64%)
Number of anti-IFN-γ auAb positive titer (%)	0/20 (0%)	22/22 (100%)	0/14 (0%)	0/40 (0%)	0/25 (0%)
Culture positive organism (%)	*M. abscessus* complex (8/20, 40%) *M. intracellulare* (3/20, 15%) *M. scrofulaceum* (2/20, 10%) *M. avium* (1/20, 5%) *M. malmoense* (1/20, 5%) *M. simiae* (1/20, 5%) *M. szulgai* (1/20, 5%) Rapidly glowing mycobacteria (1/20, 5%) Unidentified NTM (1/20, 5%)	*M. abscessus* (15/22, 68.2%) *M. intracellulare* (4/22, 18.2%) *M. kansasii* (1/22, 4.5%%) Unidentified NTM (2/22, 9.1%)	*M. tuberculosis* (14/14, 100%)	*K. pneumoniae* (11/32, 34.4%) *E. coli* (10/32, 31.3%) *P. aeruginosa* (7/32, 21.9%) *B. pseudomallei* (1/32, 3.1%) *E. coli* and *K. pneumoniae* (2/32, 6.3%) *K. pneumoniae* and *P. aeruginosa* (1/32, 3.1%)	Not determined

NTM-PD: non-tuberculous mycobacterial pulmonary disease; dNTM: disseminated non-tuberculous mycobacterial infection; MTB-PD: *Mycobacterium tuberculosis* pulmonary disease; Oth-PD: other bacterial pulmonary disease; HC: healthy control.

*P-value = 0.0097 by Kruskal–Wallis test.

**P-value less than 0.05 by Chi-square test.

In the NTM-PD group, the most common causative agent was *M. abscessus* (8/20, 40%). Similarly, the majority of patients in the dNTM group were infected with *M. abscessus* (15/22, 68.2%). All participants in the MTB-PD group tested positive for *M. tuberculosis* in sputum samples. Finally, patients with Oth-PD in this study were primarily infected with *K. pneumoniae* (11/32, 34.4%), followed by *E. coli* (10/32, 31.3%), *P. aeruginosa* (7/32, 21.9%), and *B. pseudomallei* (1/32, 3.1%).

### Effectiveness of plasma anti-GPL-core IgA in distinguishing the Thai NTM-PD group from other pulmonary infection and non-infection groups

**Figure 1:**
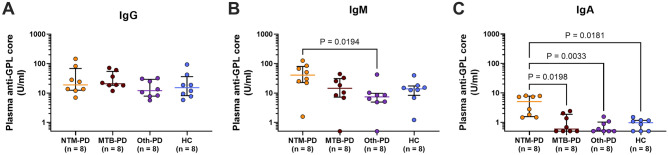
Determination of the most distinguishable plasma anti-GPL-core antibody isotype for NTM-PD. Eight plasma samples from patients with non-tuberculous mycobacterial pulmonary disease (NTM-PD), *Mycobacterium tuberculosis* pulmonary disease (MTB-PD), and other bacterial pulmonary disease (Oth-PD) and healthy controls (HCs) were quantified for anti-GPL-core antibodies using indirect ELISA. The concentration of anti-GPL-core IgM (**A**), IgG (**B**), or IgA (**C**) antibody isotypes is shown with a line representing the median and interquartile range. Statistical differences among sample groups were tested using the Kruskal–Wallis test, and only significant P-values are shown in the figure.

Our previous study demonstrated that both anti-GPL-core IgA and IgG could be used for the diagnosis of disseminated NTM (dNTM), with IgG performing better than IgA^16^. To determine the most suitable antibody isotype for Thai patients with NTM-PD, plasma levels of anti-GPL-core antibodies were measured in the first eight enrolled patients. The study quantified three plasma antibody isotypes, namely IgG, IgM, and IgA, in patients with pulmonary disease, as shown in Fig. 1. All four groups exhibited similarly high levels of plasma anti-GPL-core IgG (Fig. 1A). Conversely, the anti-GPL-core IgM isotype in the NTM-PD group was significantly higher than that in the Oth-PD group but not significantly higher than that in the MTB-PD group or healthy controls (Fig. 1B). As anticipated, the IgA isotype proved to be the most effective discriminative antibody among all pulmonary disease groups and HCs (Fig. 1C).

### Measurement of plasma anti-GPL-core IgA and IgA2 for distinguishing the Thai NTM-PD group from other pulmonary infection and non-infection groups

**Figure 2:**
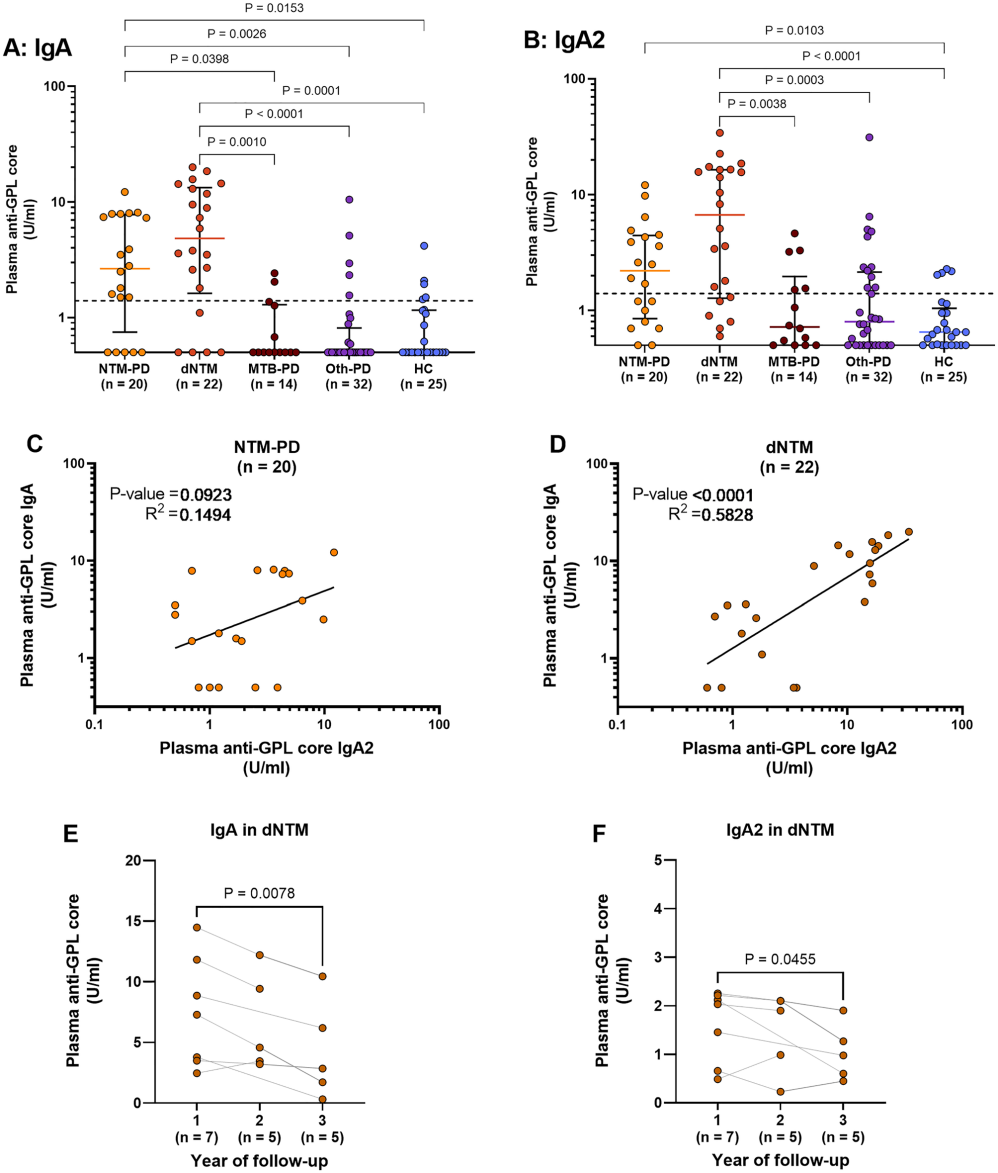
Comparison of plasma anti-GPL-core IgA and IgA2 levels among sample groups. Plasma samples from patients with NTM-PD, MTB-PD, and Oth-PD and HCs were quantified for anti-GPL-core antibodies using indirect ELISA. The concentration of anti-GPL-core IgA (**A**) or IgA2 (**B**) antibody isotypes is shown with a line representing the median and interquartile range. Statistical differences among sample groups were tested using the Kruskal–Wallis test, and only significant P-values are shown in the figure. Black dashed lines represent the cut-off values of 1.4 U/ml for IgA and 1.0 U/ml for IgA2. Correlation analysis between log_10_-transformed plasma anti-GPL-core IgA and IgA2 in patients with NTM-PD (**C**) or dNTM (**D**) was performed using linear regression. Plasma anti-GPL-core IgA (**E**) or IgA2 (**F**) antibody levels from patients with dNTM are plotted with lines connecting samples from the same patients. Antibody levels from the first sample with culture positive (1) were compared to those from the second (2) and third year of follow-up (3). Statistical significance of the decreasing antibody levels was tested using a one-tailed paired t-test, and only significant P-values are shown in the figure.

We have previously identified IgA as the most promising isotype for NTM-PD, which also suggests its potential usefulness for dNTM^16^. In this study, we analysed 113 plasma samples for anti-GPL-core IgA and its subclasses, including IgA1 and IgA2. However, we were unable to detect any signals for the IgA1 subclass (data not shown). The distribution of plasma anti-GPL-core IgA and IgA2 concentrations for each participant is presented in Fig. 2. Plasma anti-GPL-core IgA levels were significantly higher in the NTM-PD group than in the MTB-PD (P = 0.0398), Oth-PD (P = 0.0026), and HC (P = 0.0153) groups (Fig. 2A). Notably, among the 20 patients with NTM-PD, five showed negative results. Among these patients, three were infected with *M. abscessus*, one with *M. avium*, and one with *M. intracellulare*. Patients with dNTM infection exhibited significantly higher levels of plasma anti-GPL-core IgA than those with MTB-PD (P = 0.001), Oth-PD (P < 0.0001), or HC (P = 0.0001) (Fig. 2A). Four false-negative results were observed for *M. abscessus*, and one was observed for *M. kansasii*. Regarding IgA2 distribution, more interference from the controls was noted. Thus, only dNTM could be distinguished from MTB-PD (P = 0.0038), Oth-PD (P = 0.0003), and HC (P < 0.0001), while NTM-PD was significantly higher than HC (P = 0.0103) (Fig. 2B).

Furthermore, the correlation analysis between plasma anti-GPL-core IgA and IgA2 levels revealed no significant correlation in patients with NTM-PD (P = 0.0923) (Fig. 2C). Notably, patients with dNTM showed a significant association between IgA and IgA2 levels, with a correlation coefficient of R^2^ = 0.5828 (Fig. 2D). Additionally, there was no correlation between plasma anti-GPL-core IgA and anti-hIFN-γ autoantibody titres in dNTM patients (Supplementary Fig. S1).

Surplus plasma samples from seven patients with dNTM who visited the hospital annually for treatment follow-up were analysed for the decrease in anti-GPL-core levels. We observed a decrease in plasma anti-GPL-core IgA levels in the second year of follow-up (P = 0.0694), and a significant decrease was observed in the third year (P = 0.0078) (Fig. 2E). Similarly, a significant decrease in IgA2 levels was observed in the third year of follow-up (P = 0.0455) (Fig. 2F).

### Plasma anti-GPL-core IgA shows a statistically significant reduction in pulmonary infection with culture conversion at the 6-month follow-up

**Figure 3:**
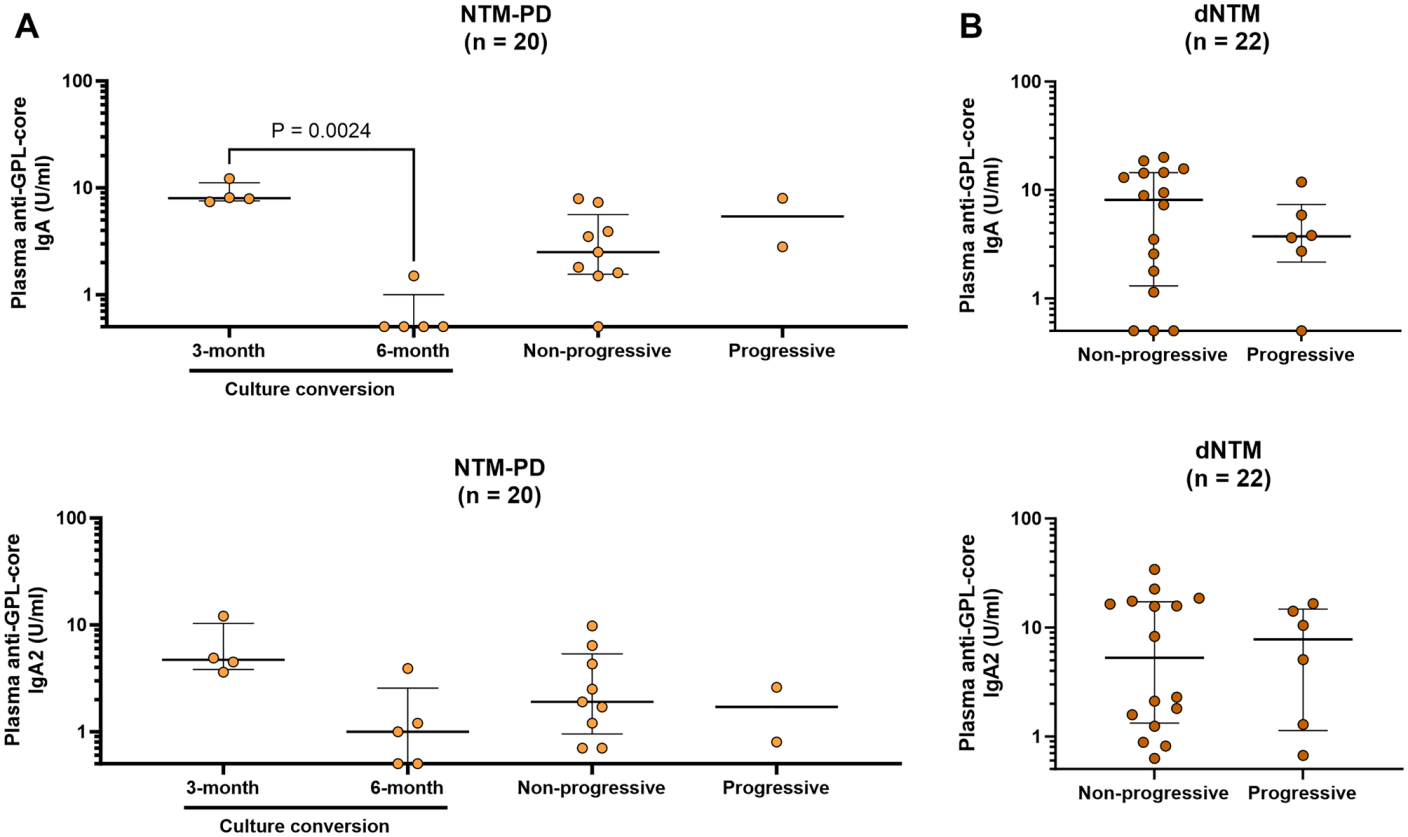
Comparison of plasma anti-GPL-core IgA or IgA2 in patients with different clinical treatment outcomes. Clinical outcomes of the patients were reviewed by clinicians. Comparisons of anti-GPL-core IgA or IgA2 were plotted as dot plots with a line representing the median and interquartile range. NTM-PD patients were subcategorised into culture conversion at the 3-month or 6-month follow-up, non-progressive, or progressive outcomes (**A**). While dNTM patients were subcategorised into non-progressive, or progressive outcomes (**B**). Statistical differences among sample groups were tested using the Kruskal–Wallis test, and only significant P-values are shown in the figure.

The clinicians reviewed the clinical data of our patients before comparing the differences in plasma anti-GPL-core antibodies among different clinical outcome groups. In the comparison of NTM-PD, a significant reduction in plasma anti-GPL-core IgA was observed in NTM-PD patients with culture conversion at the 6-month follow-up (Fig. 3A). However, NTM-PD patients with non-progressive or progressive outcome after antimicrobial treatment, exhibited similar levels of anti-GPL-core antibodies. Regarding the dNTM group, all cases included in this study were clinically active infections. When comparing anti-GPL-core antibodies between dNTM patients who were non-progressive versus progressive after treatment, no statistically significant difference was observed (Fig. 3B).

### Application of plasma anti-GPL-core IgA with a higher cut-off at 1.4 U/ml for diagnosis of Thai NTM-PD and dNTM

We analysed the diagnostic efficacy of anti-GPL-core IgA and IgA2. Applying the manufacturer's recommended cut-off of 0.7 U/ml for anti-GPL-core IgA in our specimen population resulted in 75.0% sensitivity, 69.0% specificity, 40.5% PPV, and 90.7% NPV for the diagnosis of NTM-PD (Supplementary Table S1). For the diagnosis of dNTM, the cut-off of 0.7 U/ml for anti-GPL-core IgA showed 81.8% sensitivity, 69.0% specificity, 45.0% PPV, and 92.5% NPV (Supplementary Table S1).

Receiver operating curve (ROC) analysis was conducted to determine the most appropriate cut-off level for the plasma anti-GPL-core antibody concentration. The analysis results from 20 patients with NTM-PD compared to all control groups (MTB-PD, Oth-PD, and HC; n = 71) revealed an area under the curve (AUC) of 0.788 (95% CI = 0.656–0.921) for IgA and 0.740 (95% CI = 0.616–0.864) for IgA2 (Supplementary Fig. S2A). Conversely, the analysis results from 22 patients with dNTM compared to all control groups showed an AUC of 0.848 (95% CI = 0.733–0.962) for IgA and 0.843 (95% CI = 0.753–0.932) for IgA2 (Supplementary Fig. S2B). Based on the ROC analysis for both NTM-PD and dNTM, we determined the concentration cut-off of 1.4 U/ml for IgA and 1.00 U/ml for IgA2 for further analysis.

We then applied our cut-off of 1.4 U/ml for anti-GPL-core IgA based on the ROC analysis above (Supplementary Fig. S2). The diagnostic efficacy for patients with NTM-PD (n = 20) in distinguishing them from all control groups (MTB-PD, Oth-PD, and HC; n = 71) demonstrated 75.0% sensitivity, 81.4% specificity, 53.6% PPV, and 91.9% NPV (Table 2). Similarly, patients with dNTM (n = 22) showed a sensitivity of 77.3%, specificity of 81.4%, PPV of 56.7%, and NPV of 91.9% (Table 2). With respect to the IgA2 cut-off at 1.0 U/ml, the diagnostic efficacy for patients with NTM-PD (n = 20) in distinguishing them from all control groups demonstrated 75.0% sensitivity, 63.4% specificity, 36.6% PPV, and 90.0% NPV (Table 2). Patients with dNTM (n = 22) showed 81.8% sensitivity, 63.4% specificity, 40.9% PPV, and 91.8% NPV (Table 2).

**Table 2:** Diagnostic efficacy of new cut-off values for plasma anti-GPL-core IgA and IgA2 in patients with NTM-PD or dNTM compared to all controls.

Anti-GPL-core cut-off	Infection type	Number of patients		% Sensitivity (95% CI)	% Specificity (95% CI)	% PPV (95% CI)	% NPV (95% CI)
		Positive	Negative				
IgA at 1.4 U/ml	NTM-PD	15	5	75.0% (53.1–88.8%)	81.4%* (70.8–88.8%)	53.6% (35.8–70.5%)	91.9% (82.5–96.5%)
	dNTM	17	5	77.3% (56.6–89.9%)	81.4% * (70.8–88.8%)	56.7% (39.2–72.6%)	91.9% (82.5–96.5%)
	MTB-PD	3	11				
	Oth-PD	5	27				
	HC	5	20				
IgA2 at 1.0 U/ml	NTM-PD	15	5	75.0% (53.1–88.8%)	63.4% (51.8–73.6%)	36.6% (23.6–51.9%)	90.0% (78.6–95.7%)
	dNTM	18	4	81.8% (61.5–92.7%)	63.4% (51.8–73.6%)	40.9% (27.7–55.6%)	91.8% (80.8–96.8%)
	MTB-PD	6	8				
	Oth-PD	13	19				
	HC	7	18				

PPV: positive predictive value; NPV: negative predictive value; NTM-PD: non-tuberculous mycobacterial pulmonary disease; dNTM: disseminated non-tuberculous mycobacterial infection; MTB-PD: *Mycobacterium tuberculosis* pulmonary disease; Oth-PD: other bacterial pulmonary disease; HC; healthy control.

*P-value = 0.0077 by McNemar’s Chi-Square Test.

## Discussion

According to a recent report on the prevalence of mycobacterial infections, there has been a decrease in MTB cases but an increase in NTM infections^17^. Although rare, NTM infections have been neglected. Laboratory investigations for NTM infection have poor sensitivity and are time-consuming, taking several days to months^6–8^. In this study, we evaluated the diagnostic efficacy of an anti-GPL-core ELISA kit for Thai patients with NTM-PD and dNTM. Both patient groups were confirmed as NTM culture positive. Additionally, all patients with dNTM tested positive for anti-interferon-gamma (IFN-γ) antibodies, while all patients with NTM-PD tested negative. The pathogenesis of these two types of infections differs according to the literature^18^. Anti-IFN-γ autoantibodies bind to IFN-γ and inhibit its functions, impairing phagocytosis and leading to opportunistic infections, including NTM^19^. In contrast, NTM-PD reportedly develops through different mechanisms, such as a history of MTB infection or fibrosis^18^.

Several studies on NTM infections, including NTM-PD and dNTM, have reported the most prevalent NTM species in Japan, Taiwan, and countries outside of Asia as the *Mycobacterium avium* complex (MAC), whereas in China, the Philippines, and Thailand, RGM predominates^20^. Consistent with our findings in Thai NTM-infected patients, *M. abscessus* was the most common species.

Serological diagnosis using an anti-GPL-core IgA ELISA kit has been introduced and commercialized, with a recommended cut-off of 0.7 U/ml. With this cut-off, we found a sensitivity comparable to that of previous studies but with lower specificity. Several studies in Japan have reported sensitivity and specificity above 90%^9,12^. A study in South Korea showed a sensitivity of 77.5–85% and specificity of 100%^10^. A study in Taiwan reported diagnostic efficacy with 60% sensitivity and 91% specificity^13^. Another study in the USA reported a sensitivity of 70.1% and specificity of 93.9%^11^. A more recent study in the US proposed a new cut-off of 0.178 U/ml for serological diagnosis in the American population, with 84% sensitivity, 72% specificity, 81% PPV, and 76% NPV^21^. The difference in these results may be explained by the possibility that Thai individuals have naturally developed high antibody background to endemic microbes^22^. This phenomenon has been demonstrated in a melioidosis model, where a serological test failed to diagnose owing to a high antibody background^23^.

To investigate the usefulness of serological diagnosis with anti-GPL-core antibodies for NTM infection in the Thai population, we identified the most promising antibody isotype (IgA) in patients with NTM-PD. Combined with our previous study, plasma anti-GPL-core IgA measurements showed diagnostic efficacy for patients with dNTM, with 91.18% sensitivity and 70.15% specificity^16^. ROC analysis was performed to determine the most appropriate diagnostic cut-off, resulting in 1.4 U/ml for anti-GPL-core IgA and 1.0 U/ml for IgA2. Using the new IgA cut-off, we observed a significant improvement in specificity compared to the manufacturer's recommended cut-off. Moreover, NTM-PD showed no correlation between anti-GPL-core IgA and IgA2, whereas dNTM had a very high correlation. Serum IgA and IgA2 are mainly produced from bone marrow plasma cells^24^, which are more likely to associate with systemic infection rather than localized NTM-PD. Furthermore, in the follow-up samples of patients with dNTM, anti-GPL-core IgA and IgA2 levels decreased in the second year, with statistical significance reached in the third year.

Additionally, we analysed anti-GPL-core antibodies for clinical outcomes after antimicrobial treatment in both patients with NTM-PD and dNTM. Most active infections, whether NTM-PD or dNTM, tested positive for anti-GPL-core antibodies. However, the significant reduction of anti-GPL-core IgA was observed only in NTM-PD patients with culture conversion at the 6-month follow-up, the result was in line with a previous report^25^. These data suggest the presence of anti-GPL-core antibodies persisting for 6 months after infection before declining upon clearance of the infection.

This study had several limitations, including small sample size, lack of samples with NTM colonization for comparison, lack of follow-up samples for NTM-PD, and not covering the entire spectrum of NTM infection types. HCs were enrolled using a questionnaire without laboratory investigation. Of note, the samples used in this study were heparinized plasma which were not the same as in the manufacturer’s procedure. However, in a small set of samples, the results of anti-GPL-core IgA and IgA2 from serum versus plasma samples collected from five dNTM patients showed no statistically significant difference (Supplementary Fig. S2). Due to the low sensitivity of bacterial culture, the result could not be obtained from dNTM patients during the follow-up. Moreover, we could not identify individuals with NTM colonization or infection who did not present with clinical symptoms. The patients in this study were recruited from Srinakarind Hospital, a medical hub and tertiary care centre in northeastern Thailand. Thus, this area was reportedly dominated by *M. abscessus* and MAC, with variations in their distribution among provinces^15^. Interestingly, NTM-PD patients were distinguished from other infections by IgA, but not IgA2. This implies that IgA1 may be an important subtype in NTM-PD which may require further studies. Therefore, more studies in other areas of Thailand are required to ensure the diagnostic efficacy of anti-GPL-core IgA detection.

In conclusion, this study provides an alternative serological diagnostic method for NTM infections, both pulmonary and disseminated. Using a new cut-off of 1.4 U/ml, we can improve the specificity of this test for Thai patients with a high background of antibodies while maintaining comparable sensitivity to previous studies. Additionally, previous study suggested the diagnosis of NTM-PD using single bacterial isolation combined with anti-GPL-core IgA detection^26^. Recently, the concept of using either culture independent markers or combination of traditional culture method with other markers has been reviewed to increase accuracy of diagnosis and monitoring of patients with NTM-PD or cystic fibrosis^27^. Detection of anti-GPL-core IgA should be performed to increase the rate of NTM infection detection and facilitate differentiation from MTB, enabling patients to receive more rapid and appropriate antimicrobial treatments. The association between anti-GPL-core IgA/ IgA2 and clinical outcomes has been investigated in this study, but not yet fully defined. Further studies in large clinical sample sizes are the challenges to provide more diagnostic capability of this clinical test.

## Materials and methods

### Sample enrolment and definitions

Peripheral heparinized venous blood samples were collected from adult participants (≥ 18 years of age) at Srinagarind Hospital, Khon Kaen, Thailand, between July and December 2022. The study was conducted with the approval of the Khon Kaen University Ethics Committee in Human Research (HE654007), and all participants in the NTM-PD (n = 20), *M. tuberculosis* pulmonary disease (MTB-PD; n = 14), other bacterial pulmonary disease (Oth-PD; n = 32), and healthy control (HC; n = 25) groups provided handwritten informed consent.

Patients with pulmonary disease were diagnosed by clinicians following the guidelines of the American Thoracic Society/Infectious Diseases Society of America (ATS/IDSA)^5^. Cases without immune deficiency with positive sputum cultures for *Mycobacterium tuberculosis* were classified as MTB-PD, while those with positive cultures for any *Mycobacterium* other than *M. tuberculosis* were classified as NTM-PD. Patients with positive bacterial sputum cultures for any bacteria except *Mycobacterium* were classified into the Oth-PD group. HCs were enrolled based on the blood donation guidelines of the Blood Bank of Srinagarind Hospital, Khon Kaen.

Blood sample of NTM-PD patients was collected only once. Clinical outcome at the 3-month and 6-month follow-up was reviewed by clinicians according to ATS/IDSA guidelines for monitoring of NTM disease during therapy and treatment endpoint. The goals include symptomatic, radiographic, and microbiologic improvement^5^. In more details, patients were subcategorised into (1) Culture conversion: conversion of sputum culture and/or acid fast bacilli (AFB) test to negative with symptomatic and radiographic improvement at the 3-month or 6-month follow-up, but maintained antibiotic treatment for 12 months according to the guideline, (2) Non-progressive NTM-PD: no changes in radiographic features and symptoms compared to the preceding examination with positive sputum cultures and/or AFB test, (3) Progressive NTM-PD: positive sputum cultures and/or AFB test with new radiographic abnormalities and patient presented with productive cough, fever, and dyspnea.

Surplus plasma samples from patients with dNTM and positive anti-IFN-γ autoantibody titres were obtained from routine service at Srinagarind Hospital, Khon Kaen, Thailand, between 2020 and 2022. All dNTM samples in this study were originally collected from patients with active infection who presented the signs of infection including lymphadenopathies with or without reactive skin disease, and all patients required antimicrobial drug treatment over the duration of monitoring^28^.

Treatment outcomes of dNTM on the day of sample collection were classified as (1) non-progressive dNTM group exhibited stable disease symptoms and continued receiving antimicrobial treatment without requiring hospitalization for parenteral therapy, while (2) progressive dNTM group experienced worsen clinical outcomes after treatment and required hospitalization^29^.

### Measurement of anti-GPL-core antibodies in human plasma samples

The concentration of anti-GPL-core IgA antibodies in plasma samples was measured using a GPL-core IgA ELISA kit (Capilia MAC Ab ELISA, Cat. CAMC8170, Tauns Laboratory Inc., Shizuoka, Japan) following the manufacturer's instructions.

A modification was made in the plasma dilution step for the detection of anti-GPL-core antibodies: a dilution of 1:400 for IgM and IgG subtypes, 1:40 for IgA, and 1:20 for either IgA1 or IgA2. The diluted plasma was added to the GPL-core IgA ELISA kit and incubated at room temperature (23–25 °C) for 1 h, according to the manufacturer's instructions. After washing the plates four times with the wash solution, instead of the IgA detection antibody provided in the kit, biotinylated mouse anti-human IgM (Clone G20-127, Cat. 555781, BD Biosciences) or biotinylated mouse anti-human IgG (Clone G18-145, Cat. 555785; BD Biosciences) and HRP-conjugated streptavidin (Cat. 554066; BD Biosciences) were added. For IgA1 detection, horseradish peroxidase-conjugated mouse anti-human IgA1 (Clone B3506B4, Cat. 9130-05, Southern Biotech, USA) was used, and for IgA2 detection, HRP-conjugated mouse anti-human IgA2 (Clone A9604D2, Cat. 9140-05, Southern Biotech, USA) was used. The plates were then incubated at room temperature for 1 h. After washing, the chromogen solution was added following the manufacturer's instructions, and the reaction was stopped with the stop solution. The optical density was measured at 450 nm using an ELISA reader (Tecan Magellan, Switzerland). The concentration of each antibody subtype was interpolated by comparing it with the standard curve generated from the standard samples in the kit, with a lower limit of detection of 0.5 U/ml.

### Data analysis

Statistical analyses and visualizations were conducted using GraphPad Prism version 9.5.1 (GraphPad Software, San Diego, California, USA). The normal distribution of data was assessed using the D’Agostino and Pearson tests. The Kruskal–Wallis test was employed to compare non-normally distributed data, while paired samples were compared using one-tailed paired t-tests. The chi-square test was used to assess differences in categorical data.

To analyse the sensitivity, specificity, positive predictive value (PPV), and negative predictive value (NPV), GraphPad Prism version 9.5.1 (GraphPad Software, San Diego, California, USA) was used. Statistical post hoc power analysis was conducted using MedCalc Software Ltd. McNemar's chi-squared test was utilized to examine statistical differences between the diagnostic criteria. All experiments in this study exhibited > 90% power and 95% confidence level for detecting differences between the groups.

### Supplementary Information

Supplementary Information.

## Data Availability

All data in this article are available in the paper or the supplementary material, and also are available from the corresponding author on reasonable request.
