# Supplementary material for: Evaluating anti-GPL-core IgA as a diagnostic tool for non-tuberculous mycobacterial infections in Thai patients with high antibody background
Source: Sci Rep. 2023 Nov 2;13:18883. doi: 10.1038/s41598-023-45893-8 (PMC10622420; doi:10.1038/s41598-023-45893-8)
Supplement: Supplementary file 1 — Supplementary Information. [file 41598_2023_45893_MOESM1_ESM.pdf]

Supplementary data

Evaluating Anti-GPL-Core IgA as a Diagnostic Tool for Non-tuberculous Mycobacterial Infections in Thai Patients with High Antibody Background

Varis Manbenmad[a], Apichart So-ngern[b], Ploenchan Chetchotisakd[b], Kiatichai Faksri[a], Manabu Ato[c], Arnone Nithichanon[a,*], Ganjana Lertmemongkolchai[d,*]

[a] Research and Diagnostic Center for Emerging Infectious Diseases (RCEID), Department of Microbiology, Faculty of Medicine, Khon Kaen University, Khon Kaen, Thailand

[b] Department of Medicine, Faculty of Medicine, Khon Kaen University, Thailand

[c] Department of Mycobacteriology, National Institute of Infectious Diseases, Tokyo, Japan

[d] Department of Medical Technology, Faculty of Associated Medical Sciences, Chiang Mai University, Chiang Mai, Thailand

*** Correspondence:**

Arnone Nithichanon E-mail: arnoni@kku.ac.th

Ganjana Lertmemongkolchai E-mail: ganjana.l@cmu.ac.th

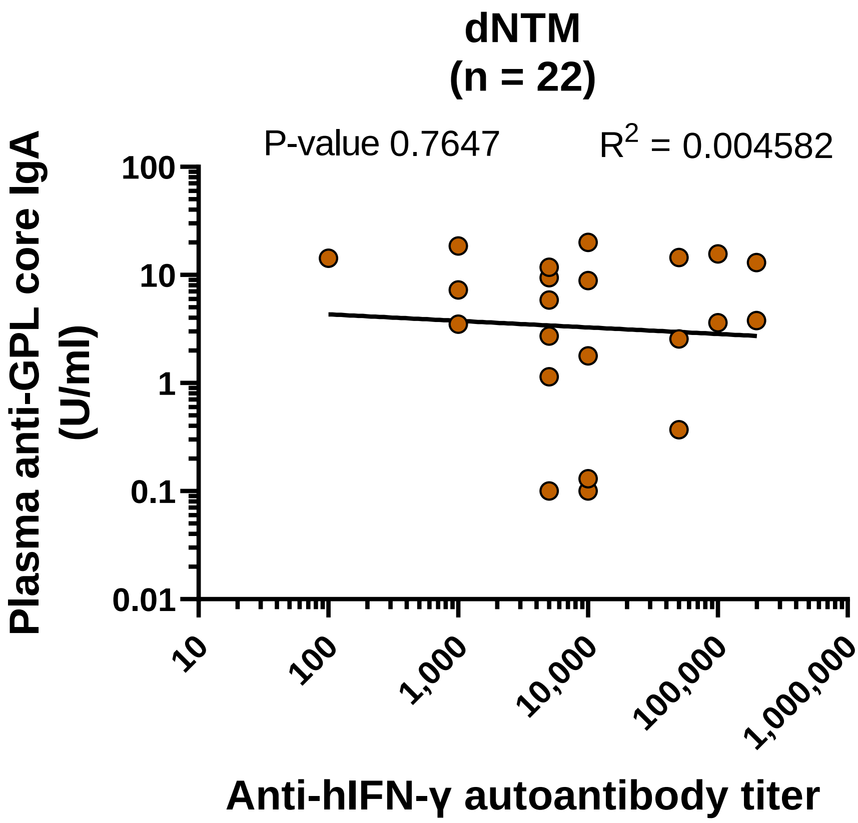

Supplementary Figure S1. Correlation between plasma anti-GPL-core IgA and anti-hIFN-γ autoantibody titers in patients with dNTM. Analysis of $\log_{10}$ transformed plasma anti-GPL core-IgA and $\log_{10}$ transformed anti-hIFN-γ autoantibody titers in patients with dNTM were performed using linear regression.

Supplementary Table S1. Diagnostic efficacies of plasma anti-GPL-core IgA for patients with NTM-PD or dNTM using cut-off value at 0.7 U/ml

Single Criteria	Infection type	Number of patients		%Sensitivity (95% CI)	%Specificity (95% CI)	%PPV (95% CI)	%NPV (95% CI)
		Positive	Negative				
**IgA at 0.7 U/ml**	NTM-PD	15	5	75.0% (53.1 – 88.8%)	69.0% (57.5 – 78.6%)	40.5% (26.4 – 56.5%)	90.7% (80.1 – 96.0%)
	dNTM	18	4	81.8% (61.5 – 92.7%)	69.0% (57.5 – 78.6%)	45.0% (30.7 – 60.2%)	92.5% (82.1 – 97.0%)
	MTB-PD	5	9				
	Oth-PD	8	24				
	HC	9	16				

PPV; positive predictive value, NPV; negative predictive value, NTM-PD; Nontuberculous mycobacterial pulmonary disease, dNTM; disseminated nontuberculous mycobacterial infection, MTB-PD; *Mycobacterium tuberculosis* pulmonary disease, Oth-PD; other bacterial pulmonary disease, HC; healthy control.

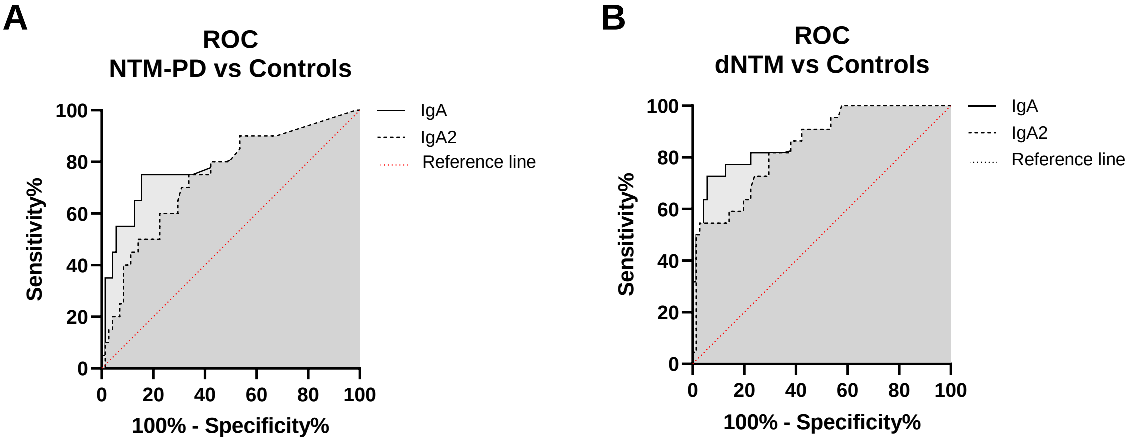

A

	AUC	SE	95% CI	P-value
**IgA**	0.788	0.068	0.656 to 0.921	<0.0001
**IgA2**	0.740	0.063	0.616 to 0.864	0.0011

B

	AUC	SE	95% CI	P-value
**IgA**	0.848	0.058	0.733 to 0.962	<0.0001
**IgA2**	0.843	0.046	0.753 to 0.932	<0.0001

Supplementary Figure S2. Receiver operating curve (ROC) analysis of IgA and IgA2 to determine the cut-off value for NTM-PD or dNTM diagnosis. Anti-GPL-core antibody levels from patients with NTM-PD **(panel A)** or dNTM **(panel B)** were plotted and compared to all control groups (MTB-PD, Oth-PD and HC). Area under curve (AUC), standard error (SE), 95% confidence interval (95% CI) and P-value were calculated according to IgA (black line) or IgA2 (black dash line) along with reference line (red dot line).

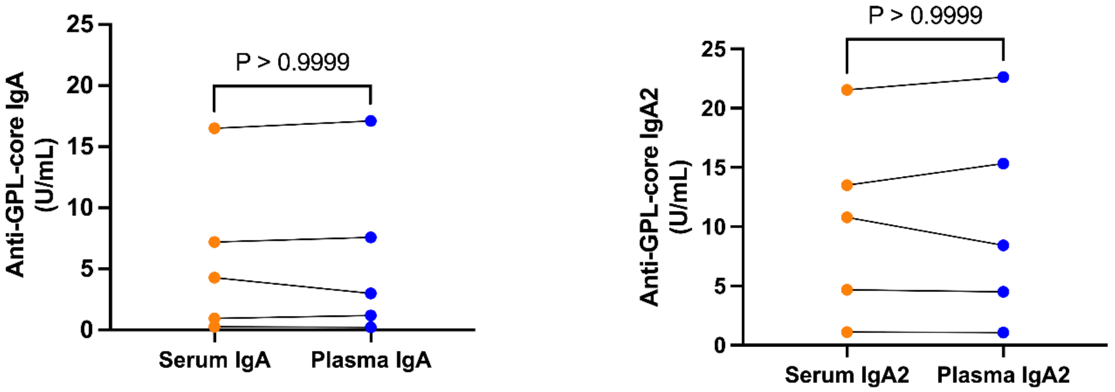

Supplementary Figure S3. Comparison of anti-GPL-core IgA and IgA2 measured from sera or plasma of the same dNTM patients (n = 5). Statistical difference was analyzed using paired t-test.
